# Consideration factors of older adults seeking medical treatment at outpatient services in Taiwan

**DOI:** 10.1186/s12913-021-07251-0

**Published:** 2021-11-09

**Authors:** Chen-I Shih, Cheng-Chie Weng, Wei Chen, Hui-Fei Yang, Sheng-Yu Fan

**Affiliations:** 1 Department of Community Health, Ditmanson Medical Foundation Chia-Yi Christian Hospital, Chia-Yi, Taiwan; 2 Department of Family Medicine, Ditmanson Medical Foundation Chia-Yi Christian Hospital, Chia-Yi, Taiwan; 3 Medical Department, Ditmanson Medical Foundation Chia-Yi Christian Hospital, Chia-Yi, Taiwan; 4 Institute of Gerontology, College of Medicine, National Cheng Kung University, No.1, University Road, 701 Tainan City, Taiwan

**Keywords:** Consideration factors for medical treatment, Health care quality, Older adults, Patient-centered

## Abstract

**Background:**

Taiwan will become a super-aged society by 2025, leading to the more frequent use of outpatient services by older adults for medical treatment compared with other age groups. Understanding the outpatient service consideration factors of older adults seeking medical treatment can improve health care quality. This study explored the selection factors and crucial considerations of older adults for outpatient services.

**Methods:**

Qualitative study was conducted. Purposive sampling was used to recruit 16 older adults over 65 years of age with chronic disease who were patients of an internal medicine department and regularly returned for checkups. Data including reasons for receiving medical treatment, factors affecting their choice of hospitals, and health care and environmental considerations were collected through structured interviews.

**Results:**

The older adults identified four factors. (1) The care of doctors: The doctors possessed professional skill, allocate sufficient consultation time, and undertake effective communication. (2) The care of other medical professionals: Other medical professionals provided services in a cordial manner. (3) The accessibility and convenience of outpatient services: Convenient transportation and registration as well as short consultation wait time. (4) Environment and equipment: The hospital had the novel facilities and satisfactory barrier-free equipment.

**Conclusions:**

The older adults cared most about the adequacy of diagnosis and treatment by doctors and other medical professionals. In addition, they reported having higher satisfaction with hospitals that provide comprehensive medical facilities, fast and convenient medical procedures, and short wait times.

## Background

In 2017, the global aging population accounted for 9 % of the total population and was expected to increase to 16 % by 2050 [1]. Taiwan became an aged society in 2019 and will become a super-aged society by 2025 [2]. Population aging exposes the need for medical services for older patients.

Health care use and expenditure among older adults are higher than those of the younger population, and the trends grow rapidly [3]. The health care use of older adults in Taiwan has escalated with the increase in the proportion of the aging population, and the number of health insurance claims for older adults increased by 26.8 % from 2007 to 2017 [4]. A total of 88 % of older adults in Taiwan have at least one chronic disease, and the five most common chronic diseases are hypertension, cataract, diabetes, cardiovascular disease, and arthritis [5]. These chronic diseases are the main cause of disease burden of older adults [6].

The medical insurance system in Taiwan is National Health Insurance, which provides low-cost but high-quality medical care [7]. The proportion of the Taiwanese population selecting medical centers, regional hospitals, and district hospitals for medical treatment is rising following an increase in the population’s age. Due to the diverse disease treatments required by older adults, primary care clinics often cannot meet their needs, and regional hospitals or higher-level medical institutions are required. Therefore, hospitals must provide care services that can meet the health needs of older adults [8].

The quality of medical services is a key factor affecting patient satisfaction. When the needs of patients are met, this affects their loyalty to the doctor [9]. The satisfaction of older adults and the choice of medical treatment are affected by various factors, including health care quality, hospital environment, and accessibility and convenience of medical treatment [10, 11]. First, health care quality is the patient’s subjective feelings about the overall medical procedure, including consultation time, doctor–patient communication, and doctor attitudes. It is highly positively correlated with overall patient satisfaction, and is also a consideration factor for patients when they choose to seek medical treatment [12, 13]. A short diagnosis and treatment time indicates less time for doctor–patient communication and psychological care for older adults, and this may affect the doctor–patient relationship [14]. Whether age prejudice is evident in the doctor’s attitude toward older adults and whether they actively provide medical care for older adults also affect the patient’s evaluation of health care quality, which in turn affects their choice of medical treatment [14]. Second, the hospital environment encompasses environmental sanitation and the clarity of environmental indicators. Older adults attach great emphasis on the environmental health and safety of hospitals, and also care about facility convenience and clear indicators enabling them to quickly and conveniently identify the location of consulting rooms [11]. Third, the accessibility and convenience of medical treatment include waiting time, online registration and payment, and feedback channels. Older adults pursue a quick, convenient medical procedure. If a hospital can provide convenient registration and online payment methods, shorten waiting time, and provide a convenient feedback channel, older adults generally provide positive feedback on overall satisfaction, and their choice of medical treatment is affected [10, 15].

Research on Taiwanese older adult outpatient health care quality and factor analysis of age-friendly policy satisfaction uncovered that factors affecting medical treatment considerations included the hospital image, quality of doctor–patient relationship, management policies, communication services, care procedures, and physical environment [16]. The hospital’s reputation and popularity can affect perceptions of the older adults. In addition to possessing adequate equipment, health care and administration should be of professional quality and must be able to effectively address patient problems. In the doctor–patient relationship, medical personnel must be able to gain the trust of older adults and protect their privacy and rights during procedures. With regard to communications, caregivers must possess excellent communication skills, provide satisfactory services with a respectful attitude and easy-to-understand language, and be able to offer preferential fees. In the care process, various evaluations (e.g., physical, psychological, medication, nutrition, economic, fall, and high risk) and care instructions must be performed according to the needs of older adults. Moreover, intervention plans should be discussed with older adults, and their right of decision should be respected. Regarding physical environments, hospitals must pay attention to safety and fall prevention, prepare barrier-free spaces and install handrails in hallways, post signs familiar to older adults, provide convenient transportation, and increase patient identification and interaction [16, 17].

Older adults are prone to multiple chronic diseases and have diverse medical care needs. They tend to visit hospitals regularly for treating and controlling their conditions. With increasing of older adults and limited resources, providing services that meet the needs of older adults is a challenge. Understanding their needs and subjective experience about outpatient services is essential to improve health care quality and patient satisfaction. Qualitative methodology can obtain detail and rich information about the process of seeking medical care. Therefore, the purposes of this study were to explore the subjective experience of older adults in outpatient and the items they valued in medical procedures.

## Methods

### Participants

 The participants were adults 65 years of age or over who were able to communicate in Mandarin or Southern Min. The inclusion criteria were normal visual and hearing function or with the correction of glasses or hearing aids, and able to communicate orally. Purposive sampling was used that sex, age, departments of clinics, and functional status were considered to keep the diversity of the participants. Older adults aged over 75 years old, with functional declines, and having chronic diseases tended to use outpatient services regularly.

The participants included both genders and three levels of age ranged 65-74, 75-84, and over 85 years old. They were patients of cardiology, metabolism, geriatrics, family medicine, gastroenterology and hepatology, and pulmonology departments. Older adults with chronic diseases in the internal medicine department who regularly returned for recurring appointments were also included. Because hearing, vision, and mobility gradually decline with age and affect the convenience of seeking medical treatment, relevant variables were included in sampling considerations. Older adults with cognitive function problems, unable to speak clearly and fluently, or reluctant to be interviewed were excluded. Regarding cognitive function, the doctors were asked to introduce older adults who did not have dementia diagnosis and had well communication ability. In the interviews, the interviewer also checked whether the participants response appropriately. For example, a participant took long time to think and could not give the answer relevant to the question directly, his or her data would be excluded in data analysis.

The recruitment location was a regional teaching hospital in southern Taiwan. The hospital provides medical care for people in the nearby counties, where 17.82 % percent of people are aged 65 and over, and the most common chronic diseases are hypertension, hyperlipidemia, and diabetes. In addition, outpatient services is one of major missions of regional teaching hospitals.

### Research design and data collection

The research team distributed the inclusion and exclusion criteria to the doctors in the relevant outpatient services who were asked to introduce the potential participants. The first author contacted the potential participants and informed the purpose and procedure of the study, and that their rights as patients would not be affected if they withdrew in the middle of the study. The participants who agreed to participate were required to sign an informed consent. This study was approved by the Institutional Review Board of the hospital (IRB number: IRB2020042).

 Structured interviews were used in consideration of the physical and cognitive abilities of the participants. However, during the interview process, the interviewer also encouraged the participants to elaborate depending on their reaction status. The interview guidelines included reasons for seeking medical treatment and hospital selection, registration procedures, transportation and parking, hospital signs and guidelines, waiting time, doctor–patient communication, circulation from consulting to examination rooms, payment, medicine dispensary, hospital environment and facilities, overall service attitude, and other recommendations. For the interview guidelines design, related studies [18–20] were referred to, and five experts including associate professors from the Institute of Gerontology, specialists in pulmonology, three outpatient nurses from the metabolism department gastroenterology and hepatology department, and family medicine department reviewed the interview outline. In addition, demographic data and audiovisual, mobility, disease, and medical information on the participants were collected. The entire interview was recorded and transcribed verbatim for analysis.

### Data analysis

Thematic content analysis [21] was conducted, and the steps are explained as follows. (1) Familiarizing with the data: repeatedly reading transcriptions and notes, and gradually developing relevant connotations and concepts through in-depth reading. (2) Generating initial codes and establishing the coding framework: the purpose of the initialization process was to mark themes mentioned by the participants, assign meaningful titles as codes, and classify and organize the codes. For example, if a participant stated that the reason for choosing a particular hospital was that the doctors are professional and reliable, the code was marked as professional competence in medicine. Moreover, all codes for professional competence in medicine were classified into one category. (3) Constructing themes: after developing the coding framework, meaningful codes related to the research question were grouped. This step was used to construct a storyline, where each theme formed part of the story. For instance, the four code categories of professional competence in medicine, doctor–patient communication, consultation time, and caring attitude formed a theme as the care of doctors. (4) Reviewing the themes: the suitability of each theme and each data segment was checked. The questions were used including: whether there were conceptual links between codes, whether codes need to be moved between themes, whether themes need to be collapsed or expanded upon, whether the meaning of each theme was described clearly, and whether the theme and its related codes told a reasonable, complete story. (5) Defining and naming themes: when naming themes, the researchers examined the meaning of each theme and clarified the key elements and core information to provide a clear, specific, and informative name to each theme.

To ensure research quality, four criteria proposed were adopted to maintain the rigor of data collection and analysis [22]. (1) Credibility: the researchers possessed qualitative research experience. In addition, the first researcher extended the interaction time before interviews to enhance mutual trust and interpersonal relationships with the participants. Moreover, they recorded the situation and ambience at the interview to increase data credibility. (2) Confirmability: interviews were recorded throughout the entire process, and the recording was typed verbatim within 72 h to reduce data omissions caused by vague memory. (3) Transferability: when collecting data, the researchers repeatedly clarified the data with participants. In addition, during analysis, the researchers recurrently examined correlations to ensure that the content responded to the research questions to ensure data completeness and extensiveness. (4) Dependability: to avoid errors in data collection resulting from different interviewers, only one interviewer (the first author) with 4 years of age-friendly promotion and research experience, including qualitative research experience, was assigned. Furthermore, the interviewer was trained to interview older adults to cultivate sensitive observation and interview skills, and data analysis capabilities. Other authors who participated in the study included geriatric case managers, doctors, and clinical psychologists, all of whom had more than 10 years of clinical experience in caring for older adults. In addition, to improve dependability, the researchers randomly selected five transcripts and reanalyzed them one month later to achieve internal consistency. The data were collected until saturation, when no new themes emerged.

The report of this study was based on Standards for reporting qualitative research (SRQR) checklist [23]. The qualitative analysis software Altasi.6.0 was employed to classify and summarize themes.

## Results

A total of 16 participants were recruited, of whom 9 were men. In terms of their age range, eight participants were 65–74 years old, five were 75–84 years old, and three were over 85 years old. In terms of education level, five were uneducated, and seven attended only elementary school. All of the participants could speak Southern Min and only those who had high school education can could speak Mandarin fluently. Six participants were patients in the metabolism department. A total of 10 participants had hearing loss, eight were presbyopic, and 12 were able to move autonomously (See Table 1). Four major themes were identified, including the care of doctors, care of other medical professionals, convenience of outpatient services, and environment and equipment (See Table 2).

**Table 1:** Demographic data of the participants

Participant code	Gender	Age	Education level	Hearing	Vision	Mobility	Frequency of visits	Department	Disease	Times of interview (minutes)
A	Male	82	Elementary school	Normal	Normal	Able to walk	Once a month	Cardiology	Hypertension	23
B	Female	92	Uneducated	Hearing loss	Normal	Wheelchair	Once a month	Cardiology	Hypertension	14
C	Female	82	Uneducated	Normal	Presbyopia	Wheelchair	Once every 3 months	Metabolism	Diabetes	20
D	Male	66	Uneducated	Hearing loss	Presbyopia	Able to walk	Twice in 3 months	Metabolism	Diabetes	21
E	Male	81	Uneducated	Hearing loss	Presbyopia	Able to walk	Once every 3 months	Metabolism	Diabetes	17
F	Male	82	High school	Hearing loss	Myopia	Able to walk	Thrice in a month	Geriatrics	Rapid weight loss	17
G	Male	69	Elementary school	Normal	Normal	Able to walk	Twice a month	Metabolism	Diabetes	29
H	Female	68	Graduate school	Normal	Presbyopia	Able to walk	Once every 3 months	Cardiology	Hypertension	26
I	Male	67	Graduate school	Normal	Myopia	Able to walk	Once every 3 months	Metabolism	Diabetes	21
J	Female	67	Elementary school	Hearing loss	Presbyopia	Able to walk	Once every 3 months	Family medicine	Diabetes	22
K	Female	70	Elementary school	Hearing loss	Myopia	Able to walk	Once every 3 months	Gastroenterology and hepatology	Gastroesophageal reflux	21
L	Female	74	Elementary school	Hearing loss	Presbyopia	Able to walk	Once every 3 months	Family medicine	Neurological disease	18
M	Male	72	Vocational school	Hearing loss	Presbyopia	Able to walk	Once every 3 months	Gastroenterology and hepatology	Gastric ulcer	22
N	Male	80	Elementary school	Hearing loss	Normal	Able to walk	Once a month	Pulmonology	Pneumonia	15
O	Male	92	Uneducated	Hearing loss	Normal	Wheelchair	When not feeling well	Gastroenterology and hepatology	Gastralgia	20
P	Female	89	Elementary school	Normal	Presbyopia	Crutch	Once every 3 months	Metabolism	Diabetes	45

**Table 2:** Overview of themes and subthemes

Theme	Subtheme	Description (numbers of coding)
Care of doctors	Professional competence in medicine	Doctors had good medical skills to solve my problems (15) I rely on and trust the doctors’ abilities (6)
	Doctor–patient communication	Doctors inquired carefully (9) Doctors had kind and genuine attitudes (5) Doctors would respect my opinions (11)
	Consultation time	There was enough consultation time (10)
	Caring attitude	Doctors had caring attitude (3) Doctors would give me personal care (4)
Care of other medical professionals	Professional care	Professional care from nurses (10), pharmacist (11), and health educators (6) were also important parts of outpatient services
	Care attitude	Care attitude from nurses (7), pharmacist (4), administration staff (10), and volunteer (3) also influenced older adults’ experience
Accessibility and convenience of outpatient services	Inconvenient transportation in rural areas	Transportation was an important concern (10), and inconvenient transportation in rural areas was one of barriers for medical care (7)
	Convenience of registration	Older adults used on-site (7), telephone (3), and internet (5) registration, and convenience of registration was an important concern (10)
	Waiting time	Older adults did not want long waiting time (8) and would like aged-priority consultation (6)
Environment and equipment	Advanced and innovative medical facilities	Older adults preferred advanced and innovative medical facilities (5)
	Excellent barrier-free equipment	Older adults needed the convenience of parking (6), the adequacy of courtesy wheelchairs (4), and the large size of signs (10)

### Theme 1: Care of doctors

In interviews, the participants spent the most time talking about the care of doctors. For them, excellent doctor care included professional competence, doctor–patient communication, consultation time, and caring attitude. Most of them cared most about health care quality, and they hoped that the doctors possessed professional competence and had sufficient time to communicate with patients for detailed diagnosis and treatment. If doctors provide medical care that meets the needs of older patients during diagnosis and treatment, and respect their opinions when formulating a care plan, older adults will have a high evaluation of the doctors’ health care quality.

Regarding the care capabilities of doctors, older adults seek medical treatment in the hope of obtaining disease treatment or symptom improvement. If the disease is improved or the symptoms are controlled after the consultation, older adults will have a high evaluation of the doctor and are willing to wait despite a long queue.

*C: I selected this hospital because the doctors are more professional and reliable, mainly because of the doctor–patient relationship.*

When receiving medical treatment, the participants cared about aspects of doctor–patient communication such as how careful and detailed the doctor’s consultation was. They hoped that doctors would explain in detail what their conditions were. The participants expected doctors to be friendly and able to interact with them and explain matters. Finally, when discussing medical treatment or making decisions, they hoped that doctors would respect their opinions.

*H: The doctor asked detailed questions and I would like to visit this doctor again next time… The doctor once wanted to increase the dose but I did not want to. I told him I will pay attention to my diet first and we shall discuss the dosing during the next visit, and he respected my opinion.*

A short consultation time (e.g., 1 to 2 min) can cause dissatisfaction in older adults. The participants were worried that they would not be able to obtain a comprehensive diagnosis and treatment if their diseases were severe, and they hoped that doctors could spend more time performing a detailed diagnosis and treatment.

*I: I would be upset if the consultation was only 1 to 2 min because it was insufficient for the doctor to check thoroughly if the disease was severe. It would be better if the doctor’s diagnosis and treatment were more detailed.*

A caring attitude is the basis of doctor care. Older adults can sense whether a doctor is caring. A caring doctor provides personalized care, such as assisting in the application if an older adult requires a special health insurance review. In addition, a caring doctor adjusts inappropriate care or medication as soon as possible, and also tracks the condition of older adults and urges them to return for medical treatment.

*K: After undergoing a surgery, my doctor asked the staff to call and remind me to go back for a checkup…The doctor is concerned about the patient.*

### Theme 2: Care of other medical professionals

Medical care is provided by a team. The participants also mentioned the care and attitudes of other medical professionals. Professionals included nurses, health educators, pharmacists, and administrators. Regarding professional care, outpatient nurses provide detailed inspection instructions and direction guidelines; health educators provide health education to returning patients with chronic diseases; and pharmacists provide medication instructions and direct for those with special needs to a medication consultation room for detailed explanations. If outpatient professionals can provide professional care in a cordial manner, older adults will have a positive experience receiving care services, which in turns generates higher satisfaction.

F: *If there is a problem with medication, the pharmacist will instruct me to go to the medication consulting room next door for consultation.*

Medical professionals are the frontline in contact with older adults, and attitude in care is an essential consideration. The participants hoped that the professionals would establish relationships, provide professional advice, and communicate patiently with a cordial, earnest, and serious attitude during the brief interactions.

*C: The nurse has a kind attitude and speaks patiently.*

### Theme 3: Accessibility and convenience of outpatient services

 In the process of seeking medical treatment, the participants were also concerned about the accessibility and convenience of outpatient services, including the transportation inconvenience in rural areas, the convenience of registration, and the wait time. The participants expected to be able to consult a doctor conveniently and quickly, and those using inconvenient transportation hoped that a hospital feeder service could be provided. In addition, they hoped that they would not need to spend a long time registering after arriving at the hospital. Moreover, they wished the wait time to be shortened as much as possible. The participants expected to receive priority access to services to increase the accessibility and convenience of outpatient services.

Although the studied hospital was located in an urban area, many older adults living in remote rural or mountainous areas visited to seek medical treatment due to the doctors’ professional competence or hospital equipment. However, they encountered transportation difficulties because Certain hospitals do not have provide feeder service for some rural areas, special vehicles to assist them, and few or no buses are available in the local area. Therefore, some participants could only travel to the hospital by taxi.

*J: Taking the bus is troublesome because I have to wait at the main road, and the bus takes half an hour to an hour to arrive.*

Regarding the convenience of registration, the participants responded that registering at the counter was inconvenient because many other patients also want to register. Sometimes doctor or department registration is full, or they receive a registration number that requires them to wait for a long period. Some participants said that they could obtain an earlier registration number in the queue through online registration, and they preferred to register online.

*H: There are numerous people registering over the counter… On-site registration can sometimes be unavailable when the number is full… Sometimes the number I get requires me to queue for a long time.*

*J: Online registration is faster, and the queue number is earlier.*

Sometimes, participants had to wait for long period because they wanted to consult a particular doctor or they required the use of particular equipment. However, they hoped to shorten the wait time and limit it to 1 h. They also hoped that older adults can be given consultation priority.

*D: I usually have to wait for a long time in large hospitals because I had to go back for a checkup… I always tell myself it is okay to wait for a while.*

*H: I hope that the wait time will not exceed 1 h.*

### Theme 4: Environment and equipment

The participants cared about regarding hospital environment and equipment including advanced, innovative facilities and excellent accessibility. For facilities and equipment, whether equipment can meet diagnosis and treatment needs affect older adult satisfaction because they attach importance to treatment effectiveness. In addition, barrier-free facilities affects the convenience of outpatient services and thus patient satisfaction. After the participants arrived at the hospital, being able to park quickly, having access to adequate courtesy wheelchairs, and clear and identifiable signs for consulting rooms were items that the participants emphasized.

Regarding medical equipment, the participants assumed that medical facilities and equipment in the teaching hospital were more complete, novel, and sophisticated than those in other hospitals, and they tended to positively evaluate the hospital’s medical equipment. Because the participants believed that the doctors and medical equipment can provide excellent health care quality on the whole, they preferred to seek medical treatment at the hospital.

*E: I chose this hospital because the doctors are better, and the machinery (medical equipment) is updated.*

Barrier-free facilities included the convenience of parking, the adequacy of courtesy wheelchairs, and the size of signs. Due to insufficient parking in the hospital and the surrounding area, the participants complained that it took a long time to obtain a parking space. Therefore, they hoped that the parking lot could be expanded or an effective parking plan be proposed to increase the accessibility and convenience of outpatient services. In addition, older adults with mobility problems often require courtesy wheelchairs, and the participants expressed that courtesy wheelchairs functioned well but were limited in number. They hoped that the hospital could purchase more wheelchairs to improve their mobility and safety. Regarding signs, the participants stated that the pictures and words were small and unclear, and they preferred to directly inquire the staff to save time. They cared about the lettering size of the sign outside the consulting room, including for the name of the doctor, the name of the nurse, and the appointment number. To avoid misreading the doctor’s name and going to the wrong room, the participants hoped that the doctor’s name could be enlarged to facilitate identification.

*P: The parking spaces are limited and it takes a long time to a find parking space… The hardest thing to identify is a parking space.*

*C: More wheelchairs should be added because they are insufficient for use…Not many wheelchairs are faulty, but their number should be increased*.

*E: The words and pictures of the signs are too small for a clear reading… I can only read the red-colored appointment number, but I cannot see the other words. …The font size of the doctor’s name should be enlarged.*

## Discussion

This study used qualitative methods to explore the crucial outpatient service consideration factors of older adults. The findings indicated that the items that concerned older adults included the care and the professional competencies of doctors and other medical professionals, the accessibility and convenience of outpatient services, environment, and equipment.

Similarly to related studies, doctor professional competency [24], doctor–patient communication, consultation time [25], and caring attitude [15] were factors that were valued by the older patients, and were crucial for improving patient satisfaction with and preference for medical treatment. The doctor competence affecting patient satisfaction included skills and the effectiveness of diagnosis and treatment. Skill quality refers to the doctor’s skill and the thoroughness and accuracy of diagnosis and treatment; the effectiveness of diagnosis and treatment refers to the assistance provided by doctors to improve or maintain patient health [26]. Older adults frequently have multiple chronic diseases and thus multiple medical needs. In interviews, they responded that doctor professional competence was the most vital item they valued. If they recognize the doctor’s competence, they are willing to wait consult the doctor despite the long queue. Furthermore, they also needed other professional to provide adequate care, for example, nurses have satisfactory skills, pharmacists offer clear medication explanations, health educators provide education according to patient needs, and the medical team has effective coordination and smooth operation.

Older adults expect to have a favorable relationship with doctors, but they may have different preferences regarding communication approaches. The participants preferred enough consultation time and that the doctor could explain information carefully and respect their decisions. The favorable doctor–patient communication included that the doctor can explain in a way that the patient can understand, show empathy and provide autonomy to the patient, this promotes [25]. Excessively short consultation time can alienate patients [14]; and when patients feel that they are being hurriedly treated during the consultation, their satisfaction with clinical experience decreases [25] as does their adherence to medical treatment [27], and the probability of medical disputes is likely to increase [28].

A caring attitude was also essential for the older adults. The caring attitude of doctors and other medical professionals are also crucial indicators of health care quality [29, 30]. Both care quality and service attitude are important for patient satisfaction. If medical professionals focus only on technical provision and care and lack psychological concern, this affects patient satisfaction and choice of outpatient services [31]. When doctors are less inclined to contact with older adults, older adults may feel isolated and not taken seriously during medical treatment [14].

The accessibility and convenience of outpatient services are critical factors in patient satisfaction and choice of outpatient services, including wait time and the convenience of transportation and registration [10, 32]. Older adults often require treatment due to the deterioration of physiological functions, and some of them had mobility difficulties. Because large hospitals are generally located in urban areas, older adults living in the countryside often face transportation problems [32]. Furthermore, Older Taiwanese adults who live in rural areas represent more than 80 % of the demand for hospital transportation. Research on the difficulties and preferences of older Taiwanese adults indicated that accessible taxis and paratransit were most popular. By contrast, public and feeder buses are not the first choice of transportation for older adults due to inconsistent schedules [33].

Regarding the convenience of registration, hospitals generally have long wait times for registration [34]. With Internet use among older adults in Taiwan increasing [35], they are beginning to use the Internet to make appointments to avoid the long wait for counter registration, and they can be informed of the number of appointments and the consultation status through real-time queue management systems. Prior studies have also noted that changing registration to a full appointment system can effectively reduce the wait time of patients and the total number of outpatients in a hospital [34]. Excessive wait time will cause patient dissatisfaction with the quality of medical services; long wait time has a significant negative correlation with patient satisfaction [24], which in turn affects the choice of outpatient services. Nonetheless, older adults are more tolerant of wait time than are younger patients. With the same wait time, older adults report a higher satisfaction, though for a wait time of more than 30 min the overall satisfaction of both young and older adults decreases [15]. Taiwan currently has an overwhelming number of outpatients, coupled with the uncertainty of the patient’s conditions; thus, the outpatient services of hospitals often encounter the problem of excessively long wait time.

In terms of equipment, they hoped that the advanced and innovative medical facilities could meet their treatment needs. In a satisfaction factor analysis of age-friendly policy, hospital image had a high correlation with novel equipment [16]. Older Taiwanese adults cared about whether the hospital provides appropriate medical equipment, which affects their willingness to revisit for medical treatment [17]. They believed advanced and innovative medical facilities could provide better care. On the other hand, as patient ages, their demand for accessible equipment also increase [17]. Accessibility is an essential consideration for older adults visiting a hospital, including the physical environment, signs and identification, transportation, and circulation [16]. Hospitals should provide specific facilities that meet the needs of older adults, such as adequate and user-friendly wheelchairs, clearly identifiable signs, and sufficient parking spaces. Convenience and accessibility are keys to improving the satisfaction and loyalty of older adults [36–38].

### Clinical applications

Doctors and other medical professionals should be intensively trained in geriatric disease and aging. In the evaluation of the older adult needs, counseling skills and training in geriatric evaluation should be provided, and sufficient time should be reserved for patient diagnosis and treatment [39]. Moreover, an interprofessional team care model for older adults should be constructed to provide comprehensive care.

Doctors and other medical professionals should improve care attitude and sensitivity to aging. They can provide patient-centered care that emphasized respecting the preferences and needs of older adults to protect the autonomy and privacy of older adults. In addition, sensitivity to aging should be improve to avoid age discrimination [40, 41].

Aged-priority clinics can be used to avoid long waits. The use of accessible taxis and paratransit can be promoted. A ride-hailing platform can be established to provide information about the start and end points, schedules, and routes of public or hospital feeder buses so as to allow multiple transportation options and promote the accessibility and convenience of outpatient services [33].

Regarding environment and equipment, excellent, innovative medical equipment is an essential medical-seeking consideration factor for older adults selecting a hospital to visit [17]. Therefore, medical equipment should be human-centric, be in line with the medical expectations and care models of the older adults, and be complete, high-performance, and modern. A clean, safe environment should also be established to reduce the risk of infection and falls. Overall, a healthy environment that complies with accessibility regulations and conforms to universal design standards should be created.

### Research limitations and recommendations

First, the data collection was conducted in an outpatient clinic in a district hospital in southern Taiwan. Although the counties where the hospital provides medical services have high proportion of older adults population, whether the results can be generalized to older adults at other locations or with other diseases requires further examination. Second, the time availability and cognitive abilities of the older adults may have affected their responses. The older adults who had cognitive function problems or could not communicate well were excluded. The included participants were mostly healthy. Future studies are recommended to include quantitative surveys and clinics or surgical departments for a more comprehensive understanding of the consideration factors of older adults regarding outpatient services. In addition, the needs of older adults with mild cognitive problems can be explored by observation or caregivers’ viewpoints.

## Conclusions

Regarding the consideration factors of outpatient services, older adults cared most about the professional competence and caring attitude of doctors, followed by the care professionalism and attitude of other medical professionals, convenient and fast medical procedures, convenient transportation, convenient registration with reduced wait times, advanced, innovative medical facilities, and barrier-free equipment. These factors can increase their satisfaction with medical services.

## Data Availability

All data generated or analysed during this study are included in this published article.
